# Sodium and potassium excretion and its association with cardiovascular disorders in Mexican adults

**DOI:** 10.3389/fnut.2024.1395016

**Published:** 2024-06-24

**Authors:** Ismael Campos Nonato, Kenny Mendoza, Jorge Vargas Meza, Mario Flores Aldana, Simón Barquera

**Affiliations:** ^1^Research Center of Nutrition and Health, National Institute of Public Health, Cuernavaca, Mexico; ^2^Department of Nutrition, Harvard TH Chan School of Public Health, Boston, MA, United States; ^3^El Poder del Consumidor A.C., Mexico City, Mexico

**Keywords:** sodium, potassium, urinary excretion, hypertension, cardiovascular disease, Mexico

## Abstract

Worldwide, as well as in Mexico, the leading cause of death is cardiovascular disease (CVD). Hypertension is the main risk factor for CVD; about 50% of the adult population suffers from this condition. High sodium (Na) intake combined with low potassium (K) intake can trigger cardiovascular disorders such as high blood pressure (BP). The aim of this study was to estimate the mean excretion of Na and K in Mexican adults using a spot urine sample, and its association with cardiovascular disorders. Information on 2,778 adults, 20–59 years of age, who participated in ENSANUT-2016 was analyzed. Na and K were estimated using Tanaka formulae. Biomarkers such as glucose, total cholesterol, triglycerides, HDL cholesterol and LDL cholesterol, and anthropometry were measured. Mean Na was 3,354 mg/day (95%CI: 3,278, 3,429), 1,440 mg/day of K (95%CI: 1,412, 1,469), and the Na-K ratio was 2.4. The excretion of Na was greater in adults with high BP (3,542 mg/day) compared to those with normal BP (3,296 mg/day). In adults with hypertension, excretion of K was 10% greater (1,534 mg/day) than in adults with normal BP (1,357 mg/day). In adults with moderate reduction of renal function, Na excretion was 22% less (2,772 mg/day) than in adults with normal kidney function (3,382 mg/day). The results of this study show that the cardiovascular health of Mexican adults is at risk, as they showed high Na excretion and low K excretion.

## 1 Introduction

Sodium (Na) and potassium (K) are essential to human homeostasis because they help in the maintenance of osmotic balance, the transmission of nerve impulses, as well as in muscle contraction or relaxation (1). In addition, these ions move against concentration gradients by the constant pumping of Na K ATP-ase in the plasma membrane of the cells. This allows three Na + ions to be expelled into the extracellular matrix and two K+ ions to enter the cytoplasm, which regulates cell volume and maintains the homeostasis of organs such as the kidney (2).

High Na intake combined with low K intake can increase vascular volume (3) and trigger vascular disorders, such as high blood pressure. Over time, this interaction between Na and K can raise blood pressure that is sensitive to salt and increase cardiovascular risk (4). It has been estimated that hypertension affects 49.4% of Mexican adults, of which 70.0% are unaware of their condition (5).

Therefore, the World Health Organization (WHO) suggests that Na consumption should be less than 2 g/day (6), while K consumption should be higher than 3.5 g/day (7). However, daily consumption of Na worldwide is 4.3 g/day (8), while K consumption is around 2.25 g/day (9). In Mexico, the national dietary intake is 3.1 g/day and 3.4 g/day of Na and K, respectively (10). In addition, a cohort study conducted by a Mexican entity, using a 24-h urine collection, estimated that 89.4% of adults have excessive Na intake, as well as inadequate K intake and Na-K ratio (11). Since the main determinant of urinary Na and K excretion is its intake, it can be determined through urine analysis.

High salt intake increases protein excretion in the urine, which causes damage to target organs: reduced kidney function, brain damage and damage to the heart; this, in turn, increases the risk of developing cardiovascular disorders (12). In addition, this excess consumption is favoring the development of stomach cancer, osteoporosis, and obesity (12). Although the gold standard calls for urine collected within 24 h, it has been shown that a spot urine sample obtained after fasting overnight can indicate daily excretion of Na and K for population studies, when using validated equations (13).

To date, there is no information that has described the average of Na and K excretion in a representative sample of adults in Mexico. Therefore, the objective of this study is to estimate mean Na and K excretion in Mexican adults participating in the National Health and Nutrition Survey 2016 (ENSANUT-2016, by its acronym in Spanish), using spot urine samples. As the Mexican population has diverse sociodemographic backgrounds and experiences a relevant burden of cardiovascular disorders influenced by dietary choices, we also aimed to obtain Na and K excretion estimates across the categories of these characteristics.

## 2 Materials and methods

### 2.1 Design and study population

The National Health and Nutrition Survey 2016 in Mexico (ENSANUT-2016) was designed to quantify the frequency and distribution of health and nutrition conditions in the Mexican population, as well as associated risk factors at the national, regional, and urban vs. rural level. ENSANUT-2016 had a transversal, probabilistic design, with regional representation (North, Center, Mexico City and South), as well as representation by area of residence, including urban (population ≥ 2,500 inhabitants) and rural (population < 2,500 inhabitants) (14). An adult ≥20 years of age was selected in each home that was visited, with a 91% response rate in 8,262 participants. Subsequently, a sub-sample of 70% of adults –aged 20 to 59 years– was selected and urine samples were collected (14).

### 2.2 Collection of sociodemographic and clinical information

Previously trained personnel applied validated questionnaires to collect sociodemographic information, personal pathological history, and lifestyle variables from participants.

### 2.3 Anthropometry

Weight and height measurements were obtained by trained personnel using international protocols (15). The body mass index (BMI) was classified according to the WHO criteria: normal BMI (18.5 to 24.9 kg/m^2^), overweight (25.0–29.9 kg/m^2^) and obesity (≥ 30.0 kg/m^2^) (15).

### 2.4 Biomarkers in urine

An 11 mL spot urine sample was collected in a conical plastic tube. The first morning urine was discarded before collection. The sample was collected at the participant’s home and was subsequently refrigerated (2–8°C) for a maximum of 7 days. Afterwards, samples were frozen at −70°C until they were processed in the National Institute of Public Health laboratory. Microalbuminuria was measured by immunoturbidimetry technique with reaction type EP -2. Creatinine was measured by the Jaffé method, and Na and K were measured through indirect potentiometry with specific electrodes for the analyte and a Na glass electrode. Na and K were estimated from the Tanaka formula (16):


$$\begin{array}{l} Estimationofsodium\left(Na\right)excretion\\ inurine\;24\;h\;(mmol/24h)=\;21.98\\ \times \left\{\begin{array}{l} \left[spoturineNa\left(mmol/L\right)/spoturineCr\left(mg/dL\right)\times 10\right]\\ \times \left[estimatedcreatinineinurine24hours\left(mg\right)\right] \end{array}\right\} \end{array}$$

$$\begin{array}{l} Estimationofpotassium\left(K\right)excretion\\ inurine\;24h\;(mmol/24h)=7.59\\ \times \left\{\begin{array}{l} \left[spoturineK\left(mmol/L\right)/spoturineCr\left(mg/dL\right)\times 10\right]\\ \times \left[estimatedcreatinineinurine24hours\left(mg\right)\right] \end{array}\right\} \end{array}$$

$$\begin{array}{l} Estimationofcreatinine\;\left(Cr\right)\;excretion\\ inurine\;\;24\;h\;\;\left(mg/24\;h\right)=-2.04\;\;\times \;\;age+14.89\;\\ \;\times \;\;weight\;\left(kg\right)+16.41\times height\left(cms\right)-2244.45 \end{array}$$

The creatinine level was measured to evaluate alterations in kidney function; it was calculated using the Chronic Kidney Disease Epidemiology Collaboration (CKD-EPI) equation, based on the Glomerular filtration rate (GFR, ml/min/1.73m^2^) (17), and categorized as follows: normal (≥90); mildly reduced (60–89); moderately reduced (30–59); severely reduced (15–29) (18).

### 2.5 Serum biomarkers

A blood sample was collected after ≥8 h of fasting. Serum biomarkers were analyzed using the Syncron® Clinical UniCel DxC 600 system. Glucose level to classify diabetes (prediabetes: fasting glucose ≥100 mg/dL and ≤ 125 mg/dL, or HbA1c ≥5.7 and < 6.5%; diagnosed diabetes: if they answered “*yes*” to the question: “*Has a doctor ever told you that you have diabetes or high blood sugar*?”; undiagnosed diabetes: if they answered “*no*” to the previous question and had fasting blood glucose ≥126 mg/dL or HbA1c ≥6.5% at the time of the survey), total cholesterol (high cholesterol levels: ≥200 mg/dL), triglycerides (high triglyceride levels: ≥150 mg/dL), hypoalphalipoproteinemia-cholesterol (HDL-c [Low HDL-c levels: women <50 mg/dL or men <40 mg/dL]), and Low-density lipoprotein-cholesterol (LDL-c [High LDL-c levels: ≥100 mg/dL]), were analyzed by endpoint coupled methods.

### 2.6 Blood pressure

Blood pressure (BP) was measured using an Omron HEM-907 XL digital sphygmomanometer following the protocol recommended by the American Heart Association (19). Adults were classified as with normal BP (systolic BP <120 mmHg and diastolic BP <80 mmHg). To identify an adult with high blood pressure, two variables were constructed: (1) diagnosed hypertension: if the adult answered “*yes*” to the question: “*Has a doctor told you that you have high blood pressure?*”; (2) undiagnosed hypertension: if the adult answered “*no*” to the previous question and they had elevated BP (systolic BP 120–129 mmHg and diastolic BP < 80 mmHg); hypertension stage 1 (systolic BP 130–139 mmHg or diastolic BP 80–89 mmHg); hypertension stage 2 (systolic BP >140 mmHg or diastolic BP >90 mmHg).

### 2.7 Ethical considerations

All participants signed the informed consent approved by the Institutional Review Board of the Mexican National Institute of Public Health (MNIPH). This study was a secondary data analysis; the Ethics and Research Commissions of the MNIPH with Commission number 1401, Bioethics registration 17 CEI00120130424, and COFEPRIS registration CEI 17007 36 approved the original protocol on March 16, 2015.

### 2.8 Data analysis

The mean, [95% confidence intervals (95%CI)] and the median [interquartile range (IQR)] of urinary excretion of Na, K, creatinine, and eGFR were computed for the overall sample and stratified by sex. Sex-based mean comparisons were performed using two-tailed t-tests, while medians were contrasted employing quantile regression models, including a binary sex indicator as the primary predictor. Subsequently, mean values (95%CIs) for Na and K excretion, as well as for the Na-K ratio, were tabulated across sociodemographic characteristics (e.g., sex, age groups, education) and cardiovascular disorders (e.g., diabetes status, dyslipidemia, renal function). Two-tailed t-tests (for binary variables) or analysis of variance (Bonferroni-adjusted ANOVA; for categorical variables with more than two groups) were utilized to compare Na excretion, K excretion, and the Na-K ratio mean values across strata of these variables. Additionally, the mean values (95%CIs) of anthropometric variables (e.g., BMI, waist circumference), blood pressure, fasting glucose, eGFR, cholesterol, HDL-c, LDL-c, and triglycerides were tabulated across urinary Na/K excretion quartiles and compared through ANOVA tests. The prevalence of diabetes status categories, hypertension strata, cardiovascular disease binary indicators, and smoking status groups was also tabulated across Na/K excretion quartiles, and comparisons were made using χ_2_ tests or Fisher exact tests. Finally, the prevalence of high sodium intake, insufficient potassium intake, and sodium–potassium ratio was calculated in the overall sample and stratified by sex. All calculations were adjusted for the complex survey design of ENSANUT using the SVY module in STATA version 14 (College Station, TX, USA).

## 3 Results

Information on 2,778 participants between 20 and 59 years of age (49.9% women and 50.1% men) was analyzed, indicating that ≈76% were overweight or obese, 46% had high blood pressure, 32% had impaired fasting glucose or diabetes, and 2% had coronary artery disease (Supplementary Table S1).

### 3.1 Distribution of concentration of the Na and K excretion

Table 1 shows that the mean urinary Na concentration was 133.6 mmol/L (95%CI: 128.7, 138.6) and that of K was 46.5 mmol/L (95%CI: 44.1, 48.9). The distribution of Na and K was not normal, so the median values for Na (129.2 mmol/L) and K (38.8 mmol/L) were estimated. When categorizing by sex, no differences were found in the average concentration of Na and K in the urine. Only a higher concentration of creatinine was observed in men (171.8 mmol/L; 95%CI: 162.9, 180.7) compared to women (142.7 mmol/L; 95%CI: 133.1, 152.3). Likewise, the mean urinary Na excretion was 3,354 mg/day (95%CI: 3,278, 3,429), mean K excretion was 1,440 mg/day (95%CI: 1,412, 1,469), and the Na-K ratio was 2.4 (95%CI: 2.3, 2.5) (Supplementary Table S2).

**Table 1:** Urinary sodium, potassium, and creatinine concentrations and eGFR of Mexican adults 20–59 years old by sex.

	Total			Men			Women		
	*n*	Mean (95%CI)	Median (IQR)	*n*	Mean (95%CI)	Median (IQR)	*n*	Mean (95%CI)	Median (IQR)
Sodium (mmol/L)	2,879	133.6 (128.7, 138.6)	129.2 (87.1, 179.0)	984	139.0 (131.3, 146.6)	132.6 (90.9, 179.5)	1,895	128.8 (122.0, 135.5)	122.7 (81.1, 170.6)
Potassium (mmol/L)	2,909	46.5 (44.1, 48.9)	38.8 (25.8, 59.3)	995	45.0 (41.9, 48.1)	37.8 (27.3, 56.1)	1,914	47.9 (44.6, 51.2)	39.9 (24.0, 65.1)
Creatinine (mmol/L)	2,883	156.5 (149.8, 163.2)	147.3 (98.6, 206.6)	978	171.8 (162.9, 180.7)	164.0 (111.8, 225.0)	1,905	142.7* (133.1, 152.3)	132.7* (88.1, 194.5)
eGFR (mL/min/1.73m^2^)^¶^	2,635	111.3 (109.9, 112.7)	112.4 (102.5, 122.6)	893	109.8 (107.9, 111.6)	110.29 (100.6, 121.8)	1,741	112.7* (110.8, 114.6)	114.4* (103.6, 123.1)

ENSANUT-2016. *This indicates a statistically significant difference between sex categories (*p* < 0.05). The mean, [95% confidence intervals (95%CI)] and the median [interquartile range (IQR)] of urinary excretion of Na, K, creatinine, and eGFR were computed for the overall sample and stratified by sex. Sex-based mean comparisons were performed using two-tailed *t*-tests, while medians were contrasted employing quantile regression models, including a binary sex indicator as the primary predictor. ^§^Estimates were adjusted for complex survey design. ^¶^Kidney function – Glomerular Filtration Rate (eGFR) was calculated according to the Chronic Kidney Disease Epidemiology Collaboration (CKD-EPI).

In obese adults, urinary Na and K excretion was ≈14% greater (Na 3,553.9 mg/day; and K 1,538.6 mg/day) than in adults with normal BMI (Na 3,113.7 mg/day; and K 1,331.1 mg/day). Excretion of Na was greater in urban and metropolitan areas –as compared to rural areas–, as well as in the northern region of the country. Concerning blood pressure, urinary excretion of Na was greater in individuals with high blood pressure stages 1 and 2 and compared to individuals with normal blood pressure or who had a previous diagnosis of hypertension. Furthermore, excretion of Na was greater in individuals with prediabetes and those who were found to have type 2 diabetes, compared to those with normal fasting glucose levels and those previously diagnosed with type 2 diabetes (Supplementary Table S2).

Na excretion was lower in individuals with a hypercholesterolemia diagnosis and elevated LDL-c, compared to individuals with normal cholesterol and LDL-c. However, it was higher in those with hypoalphalipoproteinemia (low HDL-c) compared to individuals who had normal HDL-c. Na excretion was positively associated (higher) in individuals diagnosed with cerebrovascular disease and coronary artery disease (Supplementary Table S2).

Urinary K excretion was positively associated with older age (50–59 years), SES, the northern region of the country, and BMI. Likewise, a positive trend was observed in relation to K excretion and blood pressure, which was higher in individuals who had a previous diagnosis of hypertension and type 2 diabetes, as well as those who were found to have type 2 diabetes and prediabetes through the survey, as compared to those with normal fasting glucose levels (Supplementary Table S2).

Greater K excretion was observed in individuals with a diagnosis of cerebrovascular disease, compared to adults without this condition. In adults diagnosed with hypertension, K excretion was 10% higher (1,534 mg/day; 95%CI: 1,470, 1,599) than in adults with normal blood pressure (1,357 mg/day; 95%CI: 1,357, 1,435). In relation to adults with moderate reduction of renal function (MRFR), Na excretion was 22% lower (2,772 mg/day; 95%CI: 2,300, 3,244) than in adults with normal renal function (3,382 mg/day; 95%CI: 3,301, 3,462). Likewise, the Na-K ratio was 2.1 in adults with MRFR and 2.4 in adults with normal kidney function (Supplementary Table S2).

### 3.2 Na and K excretion: nutrition status, clinical characteristics, and health risk

When categorizing urinary Na excretion in quartiles (Table 2), it was observed that the mean waist circumference was greater in women (97.8 cm) and men (99.5 cm) in the fourth quartile (4,436 mg/day) than in the first quartile of Na (2,291 mg/day), being 90.7 cm in women and 90.5 cm in men. In the fourth quartile of K (1,948 mg/day), the mean BMI was higher (29.6 kg/m^2^) than in the first quartile of K (1,030 mg/day and BMI of 26.6 kg/m^2^) (Table 3). The values for mean waist circumference in men and women, as well as for hypertension diagnosis, were positively associated with a higher urinary excretion of K (first quartile to fourth quartile) (Table 3).

**Table 2:** Nutritional status and clinical characteristics according to quartiles of urinary sodium excretion in the Mexican adults’ population.

Urinary sodium excretion quartiles (mg/day)	*n*	First	Second	Third	Fourth
		Mean (95%CI)	Mean (95%CI)	Mean (95%CI)	Mean (95%CI)
		2,330.8 (2,291.3, 2,370.4)	2,993.3 (2,965.4, 3,021.1)	3,525.3 (3,503.9, 3,546.7)	4,436.7 (4,321.1, 4,552.3)
BMI (kg/m^2^)^a^	2,768	26.8 (26.2, 27.4)	28.0* (27.4, 28.7)	29.1* (28.4, 29.8)	29.8* (28.7, 30.9)
Waist circumference (cm)					
Women	1,730	90.7 (86.3, 95.2)	91.5 (88.8, 94.2)	96.3* (93.9, 98.7)	97.8* (96.0, 99.5)
Men	943	90.5 (88.3, 92.7)	94.0* (91.5, 96.6)	97.8* (95.2, 100.4)	99.5* (96.0, 103.1)
SBP (mm Hg)	2,709	115.7 (113.3, 118.1)	115.6 (113.0, 118.2)	117.5 (115.3, 119.7)	116.5 (112.4, 120.6)
DBP (mm Hg)	2,709	72.6 (71.0, 74.3)	72.7 (70.9, 74.6)	74.4 (72.9, 75.8)	73.0 (71.3, 74.6)
eGFR (mL/min/1.73 m^2^)	2,563	111.6 (107.0, 116.2)	113.7 (109.9, 117.5)	117.5 (112.9, 122.1)	116.7 (111.9, 121.4)
Glucose (mg/dL)	2,701	103.6 (97.8, 109.5)	97.7 (94.7, 100.7)	102.1 (97.4, 106.7)	100.5 (96.6, 104.5)
Cholesterol (mg/dL)^b^	2,568	196.6 (185.1, 208.2)	190.1 (184.1, 196.1)	185.3 (179.1, 191.6)	186.7 (179.9, 193.6)
HDL-c (mg/dL)^c^	2,568	40.8 (39.1, 42.6)	40.8 (37.4, 44.3)	38.5 (36.9, 40.2)	36.9* (35.5, 38.4)
LDL-c (mg/dL)^d^	2,387	120.6 (109.9, 131.4)	114.7 (109.6, 119.7)	108.7* (103.8, 113.6)	111.3 (101.4, 121.1)
TG (mg/dL)^e^	2,568	188.1 (172.7, 203.5)	186.1 (169.2, 203.0)	194.9 (178.3, 211.5)	220.1 (190.9, 249.3)
Diabetes^f^ (%)	2,478				
Prediabetes		18.2 (13.8, 23.6)	18.8 (14.5, 24.1)	22.2 (16.5, 29.2)	26.5 (18.3, 36.8)
Diagnosed diabetes		7.7 (4.5, 12.8)	4.0 (2.6, 6.0)	6.1 (4.1, 9.0)	5.9 (3.9, 8.9)
Undiagnosed diabetes		2.8 (1.6, 4.9)	2.8 (1.5, 5.0)	2.5 (1.3, 4.8)	4.1 (1.7, 9.4)
High blood pressure^g^ (%)	2,709				
Elevated		9.2 (6.1, 13.6)	6.0 (4.2, 8.5)	10.2 (6.9, 15.0)	10.8 (7.2, 16.0)
Stage 1		15.2 (8.7, 25.3)	15.5 (11.5, 20.5)	24.5 (18.5, 31.8)	16.7 (11.8, 23.1)
Stage 2		7.5 (3.8, 14.4)	6.6 (3.7, 11.5)	8.3 (4.3, 15.3)	7.7 (4.6, 12.5)
Diagnosed hypertension		9.1 (6.1, 13.4)	10.6 (6.1, 17.8)	8.2 (5.7, 11.8)	6.9 (4.6, 10.2)
Coronary Heart Disease (%)	2,756	0.8 (0.3, 2.0)	1.3 (0.4, 4.0)	1.4 (0.6, 3.2)	2.4 (1.0, 5.8)
Cerebrovascular disease (%)	2,729	0.1 (0.1, 0.3)	0.6 (0.2, 1.4)	0.2 (0.1, 0.7)	0.7 (0.2, 1.8)
Smoking (%)	2,752				
Never-smoker		40.3 (31.5, 49.8)	43.7 (35.6, 52.2)	48.4 (41.4, 55.4)	44.7 (33.6, 56.4)
Current smoker		25.7 (16.3, 38.0)	17.8 (12.3, 24.9)	19.9 (14.8, 26.2)	22.1 (16.2, 29.4)
Ex-smoker		34.1 (27.2, 41.8)	38.5 (31.5, 46.0)	31.8 (26.1, 38.0)	33.2 (24.4, 43.3)

ENSANUT-2016. Estimates were adjusted for complex survey design. *This indicates a statistically significant difference between first quartile and other quartiles (p < 0.05). Mean values (95% CIs) of variables were tabulated across urinary Na excretion and K excretion and compared using ANOVA tests. ^a^Body mass index (BMI): <25 kg/m^2^ (normal); 25–29.9 kg/m^2^ (overweight); ≥30 kg/m^2^ (obesity). ^b^High total cholesterol levels: ≥200 mg/dL. ^c^Low HDL-c levels (hypoalphalipoproteinemia): women < 50 mg/dL or men < 40 mg/dL. ^d^High LDL-c levels: ≥100 mg/dL. ^e^High triglycerides levels: ≥150 mg/dL. ^f^Diabetes classification: prediabetes: fasting glucose ≥ 100 mg/dL and ≤ 125 mg/dL, or HbA1c ≥ 5.7 and < 6.5%; diagnosed diabetes: if they answered “yes” to the question: “Has a doctor ever told you that you have diabetes or high blood sugar?”; undiagnosed diabetes: if they answered “no” to the previous question and had fasting blood glucose ≥ 126 mg/dL or HbA1c ≥ 6.5% at the time of the survey). ^g^Blood pressure (mm Hg): normal (<120/80); elevated (systolic between 120–129 and diastolic < 80); stage 1 (systolic between 130–139 or diastolic between 80–89); stage 2 (systolic at least 140 or diastolic at least 90. Diagnosed hypertension was defined when the adult answered “yes” to the question: “Has a doctor told you that you have high blood pressure?”.

**Table 3:** Nutritional status and clinical characteristics according to quartiles of urinary potassium excretion in the Mexican adults’ population.

Urinary potassium excretion quartiles (mg/day)	*n*	First	Second	Third	Fourth
		Mean (95%CI)	Mean (95%CI)	Mean (95%CI)	Mean (95%CI)
		1,030.8 (1,015.8, 1,045.8)	1,278.3 (1,265.9, 1,290.6)	1,521.5 (1,505.7, 1,537.4)	1,948.1 (1,913.6, 1,982.7)
BMI (kg/m^2^)^a^	2,794	26.6 (26.1, 27.2)	27.7* (26.8, 28.7)	30.0* (29.2, 30.7)	29.6* (29.0, 30.2)
Waist circumference (cm)					
Women	1,748	88.5 (86.4, 90.5)	92.3* (89.9, 94.7)	98.0* (94.1, 101.9)	97.9* (96.3, 99.4)
Men	952	90.4 (88.2, 92.7)	92.6 (90.8, 94.5)	100.8* (98.2, 103.5)	99.6* (97.0, 102.3)
SBP (mm Hg)	2,736	114.7 (112.5, 116.8)	113.7 (110.0, 117.3)	118.5* (116.3, 120.6)	119.0* (116.3, 121.6)
DBP (mm Hg)	2,736	71.9 (70.6, 73.1)	72.4 (70.5, 74.3)	74.31* (73.03, 75.58)	74.1* (72.6, 75.6)
eGFR (mL/min/1.73 m^2^)	2,563	109.1 (105.7, 112.5)	111.2 (108.9, 113.4)	112.1 (109.3, 114.8)	112.7 (110.4, 115.0)
Glucose (mg/dL)	2,728	96.8 (94.4, 99.2)	100.8 (95.4, 106.3)	101.8* (97.4, 106.2)	104.4* (100.0, 108.7)
Cholesterol (mg/dL)^b^	2,593	180.0 (173.1, 186.9)	189.2 (180.9, 197.6)	195.5* (185.5, 205.6)	192.3* (186.6, 198.0)
HDL-c (mg/dL)^c^	2,593	39.5 (36.0, 43.1)	39.7 (37.9, 41.5)	38.2 (36.9, 39.5)	39.46 (37.79, 41.13)
LDL-c (mg/dL)^d^	2,412	107.5 (102.4, 112.6)	113.9 (105.2, 122.6)	117.5 (106.0, 128.9)	115.0* (109.5, 120.5)
TG (mg/dL)^e^	2,593	182.7 (164.2, 201.2)	184.8 (167.4, 202.2)	218.1* (201.9, 234.3)	206.2 (187.6, 224.8)
Diabetes^f^ (%)	2,502				
Prediabetes		19.9 (15.0, 26.0)	17.6 (12.5, 24.2)	24.5 (19.1, 30.8)	24.2 (18.4, 31.1)
Diagnosed diabetes		3.8 (2.1, 6.7)	5.1 (2.8, 8.9)	8.0* (5.4, 11.7)	8.0* (5.5, 11.6)
Undiagnosed diabetes		2.1 (1.1, 4.1)	3.7 (2.2, 6.4)	1.9 (1.0, 3.6)	4.5 (1.9, 10.3)
High blood pressure^g^ (%)					
Elevated	2,736	10.3 (6.7, 15.5)	8.9 (5.9, 13.1)	6.3 (4.3, 9.3)	11.1 (7.4, 16.3)
Stage 1		17.5 (12.1, 24.5)	10.2* (6.9, 14.7)	24.3 (17.4, 32.9)	21.1 (15.8, 27.6)
Stage 2		4.4 (2.6, 7.3)	6.9 (3.8, 12.1)	9.7 (5.6, 16.5)	9.5 (5.4, 16.2)
Diagnosed hypertension		4.4 (2.7, 7.1)	7.8 (4.7, 12.6)	10.5* (7.4, 14.5)	11.7* (8.0, 16.7)
Coronary Heart Disease (%)	2,783	1.6 (0.5, 4.7)	1.6 (0.5, 4.7)	1.3 (0.6, 2.6)	1.5 (0.6, 3.9)
Cerebrovascular disease (%)	2,755	0 (0)	0.6 (0.2, 1.6)	0.5 (0.2, 1.2)	0.3 (0.1, 1.1)
Smoking (%)	2,779				
Never-smoker		46.0 (38.2, 54.0)	47.4 (35.9, 59.2)	40.7 (33.4, 48.4)	41.5 (34.2, 49.2)
Current smoker		21.3 (15.9, 28.0)	21.8 (13.9, 32.6)	22.3 (13.7, 34.1)	20.2 (14.6, 27.2)
Ex-smoker		32.7 (26.6, 39.4)	30.7 (22.4, 40.5)	37.0 (30.4, 44.2)	38.4 (31.8, 45.5)

ENSANUT-2016. Estimates were adjusted for complex survey design. *This indicates a statistically significant difference between first quartile and other quartiles (*p* < 0.05). Mean values (95% CIs) of variables were tabulated across urinary Na excretion and K excretion and compared using ANOVA tests. ^a^Body mass index (BMI): <25 kg/m^2^ (normal); 25–29.9 kg/m^2^ (overweight); ≥30 kg/m^2^ (obesity). ^b^High total cholesterol levels: ≥200 mg/dL. ^c^Low HDL-c levels (hypoalphalipoproteinemia): women < 50 mg/dL or men < 40 mg/dL. ^d^High LDL-c levels: ≥100 mg/dL. ^e^High triglycerides levels: ≥150 mg/dL. ^f^Diabetes classification: prediabetes: fasting glucose ≥ 100 mg/dL and ≤ 125 mg/dL, or HbA1c ≥ 5.7 and < 6.5%; diagnosed diabetes: if they answered “yes” to the question: “Has a doctor ever told you that you have diabetes or high blood sugar?”; undiagnosed diabetes: if they answered “no” to the previous question and had fasting blood glucose ≥ 126 mg/dL or HbA1c ≥ 6.5% at the time of the survey). ^g^Blood pressure (mm Hg): normal (<120/80); elevated (systolic between 120–129 and diastolic < 80); stage 1 (systolic between 130–139 or diastolic between 80–89); stage 2 (systolic at least 140 or diastolic at least 90. Diagnosed hypertension was defined when the adult answered “yes” to the question: “Has a doctor told you that you have high blood pressure?”.

**Figure 1:**
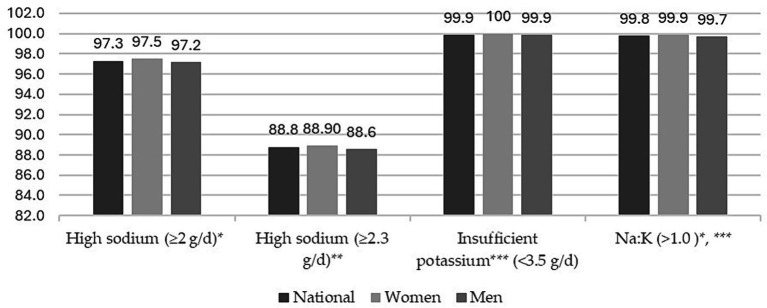
Proportions (%) of high sodium, insufficient potassium intake, and sodium–potassium ratio by sex in Mexican population. ENSANUT-2016.

Figure 1 shows the proportion of participants with high Na and K intake, as well as a Na-K ratio greater than 1.0. About 97% of the adult population intakes Na above the WHO recommendations. However, the proportion of the adult population that exceeds the Na intake recommended by the AHA is 89%. Nevertheless, the Mexican adult population has insufficient K intake in accordance with WHO recommendations. Likewise, the entire Mexican adult population has a Na-K ratio > 1. No differences by sex were found.

## 4 Discussion

In this nationwide representative sample of 2,778 Mexican adults aged 20 to 59 years, a mean urinary Na excretion of 133.6 mmol/L and K excretion of 46.5 mmol/L was observed, with a Na-K ratio of 2.4. In addition, urinary Na excretion was higher in individuals with stage 1 and stage 2 hypertension, in individuals with prediabetes, and in those found to have type 2 diabetes, whereas it was positively associated in individuals diagnosed with cerebrovascular disease and coronary heart disease; whereas Na excretion was lower in individuals diagnosed with hypercholesterolemia and elevated LDL-c compared to individuals with normal cholesterol and LDL-c. Likewise, higher K excretion was observed in individuals with a previous diagnosis of hypertension and type 2 diabetes, as well as in those with type 2 diabetes and prediabetes detected through the survey, cerebrovascular disease, and adults with a diagnosis of hypertension.

In this study, Na and K intakes were slightly lower according to what was found in a systematic review in America, where they found mean excretion of 157.3 mmol/24 h for Na and 57.7 mmol/24 h for K (20). Likewise, in Latin America and the Caribbean, it has been identified that the mean Na intake is close to 149.13 mmol/L, which is like that identified in this study, but lower than the estimates for Colombia, Brazil, and Chile (204.4 mmol/L) (21). In Mexico, estimates from diet are very similar to those obtained in this study where a urine spot sample was obtained (3.1 g/day vs. 3.4 g/day, respectively) (10).

These Na and K excretion values were lower than those observed in a study conducted in more than 100,000 individuals from 17 low-and middle-income countries (4,930 and 2,120 mg/day, respectively) (22). However, the median Na excretion in the present study (3,315 mg/day) would reflect a Na consumption that far exceeds the American College of Cardiology/American Heart Association recommendations of 1,500–2,400 mg/day. Thus, more than 50% of Mexican adults would have an excessive Na intake according to these recommendations (23).

Nonetheless, the WHO and the Pan American Health Organization (PAHO) have established a goal to reduce Na consumption in adults to less than 2 grams (<87 mmol) per day by 2025 (6, 24). On the other hand, various organisms recommend K consumption to be above 3.5 grams per day (≥90 mmol). The data from our study indicate that more than 90% of Mexican adults exceed the Na recommendation and fail to meet the K recommendation (7).

Urinary Na excretion was higher in individuals with elevated BP and hypertension stages 1 and 2, compared to individuals with normal BP or who had a previous diagnosis of hypertension. Likewise, Na excretion was higher in individuals with prediabetes and those found to have diabetes type 2 through the survey, compared to individuals with normal fasting glucose and those previously diagnosed with diabetes type 2. Na excretion was lower in individuals with a hypercholesterolemia diagnosis and elevated LDL-c, compared to individuals with normal cholesterol and LDL-c. These findings possibly reflect a phenomenon of “reverse causality” and point to a possible modification of dietary habits in individuals who know their clinical condition (25). However, Na excretion was higher in individuals diagnosed with cerebrovascular disease and heart disease, which possibly translates to poor dietary control in these patients. Likewise, a review study showed that decreased sodium intake increases serum cholesterol, as well as HDL-c and LDL-c concentrations (26).

The positive association of Na excretion with male sex, BMI, residence in urban and metropolitan areas, socioeconomic level, and being in the northern region of the country possibly reflects a higher intake of high-sodium foods (for example, highly processed foods) (27) and low fruit and vegetable intake (28). In turn, these are associated with unhealthy dietary patterns that are conducive to chronic degenerative diseases (29).

When a person is diagnosed with high blood pressure, the first strategy for treatment and prevention of complications is to restrict sodium intake to less than 2,300 mg/day (30). This can coexist with the loss of K and consequently develop muscle weakness, irregular heart rhythm and increased sensitivity to salt (31). However, in our study, individuals with a previous diagnosis of hypertension (HT) had higher K excretion than individuals with normal blood pressure.

In the past three decades, elevated BP has been the main risk factor for preventable deaths (32). In Mexico, mortality due to high BP has increased by 29.9% in the past six years and in 2016 it was responsible for 18.1% of all deaths (33). It has been shown that daily excretion of Na and K can be used to estimate intake; nonetheless, measuring these electrolytes in urine samples collected within 24 h poses practical limitations for use in population studies. For this reason, different formulae have been developed to estimate the daily intake of Na and K from spot urine samples. Among the most used are the INTERSALT, Kawasaki, and Tanaka formulae, since these have achieved high precision when compared with the reference method (13). This last method, which we applied in our study, has a correlation of 0.72 for Na and 0.78 for K, and has shown to be potentially useful in studies with many individuals (34).

Diverse studies show a direct association between reduced consumption of Na and lower BP. In this sense, the lower sodium consumption is, the lower the cardiovascular risk (35). On the other hand, there is strong evidence of the association between an increase in K consumption and a linear reduction in BP and CVD (36). Since these minerals are closely related to BP and CVD, calculating the Na-K ratio is recommended. A ratio of osmolality of Na-K ratio < 1 in urine can be a useful indicator to meet levels of Na and K recommended by the WHO, as well as to reduce hypertension and CVD risk (3, 36, 37). It has been shown that a Na-K ratio of 1.13 (95%CI: 1.04, 1.22) for coronary heart disease and 1.20 (95%CI: 1.01, 1.42) for heart failure can cause a risk of 1.11 (95%CI: 1.04, 1.19) for a combined outcome of cardiovascular disease (37–39).

In the last decades, eating patterns in Mexico have shifted toward increased consumption of ultra-processed foods, accompanied by a reduction in fruit and vegetable intake (40). Therefore, the modern diet is high in Na, sugar, and fat content, but low in fiber (41). In addition, processed and ultra-processed products contribute, on average, 46% of the total Na in the diet, whereas minimally processed foods contribute more than 50% of the intake of K (10). In parallel, about 49% of Mexican adults have hypertension and the main cause of death is cardiovascular disease (5). If this trend continues, the Na-K ratio could increase, and this would have an impact on increased cardiovascular risk (42). Simulation studies have estimated that reducing Na consumption in Mexico as recommended by the WHO could reduce around 27,700 deaths from cardiovascular diseases, mainly ischemic heart disease, hypertensive disease, and stroke (43).

Among the strengths of the study are its representativeness, sample size, and the laboratory methods used. On the other hand, concerning the limitations of the present study, we cannot fail to mention the inherent difficulty of establishing causal association—for example, when evaluating the association between Na excretion and metabolic alterations, given that individuals with a previous diagnosis had lower levels compared to individuals who were assumed to be healthy or were unaware of their condition. This problem of reverse causality is common to several types of studies, especially cross-sectional ones (44). Another limitation, which is inherent to the use of the urine spots technique for population-level research because of its low risk of error and lower collection burden, and because it is more affordable than the collection of 24-h urine, is that these equations have been shown to be an inaccurate estimate of Na intake of 24-h dietary recall (45) and can overestimate low intake and underestimate high intake (45, 46). Additionally, the measurement of sodium in a single urine sample is affected by the salt content of recently ingested foods and diurnal excretion patterns. Therefore, we recognize that the association between sodium intake and blood pressure would likely have been improved if multiple urine samples had been measured rather than just the one (47) that we obtained in our study.

On the other hand, the excretion of K measured in urine is less precise than that of Na and represents approximately 80% of the intake. Underestimating the intake of K will lead to an overestimation of the proportion of individuals with low intake (47).

## 5 Conclusion

Until now, Mexico had not had representative population data on urinary Na and K excretion. The results of this study will provide an estimate of Na and K intake in the Mexican adult population.

The present study, performed in a representative sample of Mexican adults, shows high Na excretion and relatively low K excretion, in accordance with international recommendations aimed at reducing cardiovascular risk. However, concrete actions are required at the public policy level to reduce sodium intake in the Mexican population. Nevertheless, this information can serve as a reference for public policies aimed at controlling arterial hypertension and reducing CVD.

## Data availability statement

The data that support the findings of this study are available from the corresponding author upon reasonable request. The National Health and Nutrition Survey (ENSANUT, by its Spanish acronym) database used in this study is publicly available and can be accessed at https://ensanut.insp.mx/.

## Ethics statement

The studies involving humans were approved by the Institutional Review Board of the National Institute of Public Health in Mexico. This study was a secondary data analysis; Ethics and Research Commissions of the MNIPH with the Commission number 1401, Bioethics registration 17 CEI00120130424, and COFEPRIS registration CEI 17007 36 approved the original protocol. The studies were conducted in accordance with the local legislation and institutional requirements. The participants provided their written informed consent to participate in this study.

## Author contributions

IC: Conceptualization, Funding acquisition, Investigation, Supervision, Validation, Visualization, Writing – original draft, Writing – review & editing. KM: Conceptualization, Data curation, Formal analysis, Investigation, Methodology, Software, Writing – review & editing. JV: Formal analysis, Methodology, Validation, Visualization, Writing – review & editing. MF: Writing – original draft, Writing – review & editing, Conceptualization, Investigation. SB: Validation, Visualization, Writing – review & editing.
