# Supplementary material for: Sodium and potassium excretion and its association with cardiovascular disorders in Mexican adults
Source: Front Nutr. 2024 Jun 24;11:1395016. doi: 10.3389/fnut.2024.1395016 (PMC11228292; doi:10.3389/fnut.2024.1395016)
Supplement: Supplementary file 1 [file Table_1.DOCX]

**Supplementary Table 1.** Characteristics of Mexican adults 20-59 years by sex. ENSANUT-2016.

|  | **Total** | | | | **Women** | | | | **Men** | | | |
| --- | --- | --- | --- | --- | --- | --- | --- | --- | --- | --- | --- | --- |
|  | **n** | **N** | **%** | **95%CI** | **n** | **N** | **%** | **95%CI** | **n** | **N** | **%** | **95%CI** |
| **National** | 2778 | 52,666 | - | - | 1821 | 26,278 | 49.9 | 46.2, 53.6 | 957 | 26,388 | 50.1 | 46.4, 53.8 |
| **Age (years)** |  |  |  |  |  |  |  |  |  |  |  |  |
| 20-29 |  |  | 28.8 | 25.7, 32.2 |  |  | 28.0 | 24.4, 31.9 |  |  | 29.6 | 24.7, 35.1 |
| 30-39 |  |  | 29.9 | 26.5, 33.6 |  |  | 27.9 | 23.9, 32.3 |  |  | 31.9 | 26.7, 37.7 |
| 40-49 |  |  | 22.0 | 19.4, 24.9 |  |  | 24.2 | 20.9, 27.8 |  |  | 19.9 | 16.4, 24.04 |
| 50-59 |  |  | 19.3 | 16.6, 22.1 |  |  | 19.9 | 16.3, 24.1 |  |  | 18.6 | 14.9, 22.7 |
| **Socioeconomic index** | 2778 | 52,666 | - | - | 1821 | 26,278 | 49.9 | 46.2, 53.6 | 957 | 26,388 | 50.1 | 46.4, 53.8 |
| Low |  |  | 22.5 | 19.7, 25.7 |  |  | 22.1 | 19.1, 25.6 |  |  | 22.9 | 19.1, 27.3 |
| Medium |  |  | 31.1 | 28.0, 34.4 |  |  | 30.1 | 26.4, 33.9 |  |  | 32.2 | 27.5, 37.1 |
| High |  |  | 46.4 | 42.3, 50.5 |  |  | 47.8 | 43.3, 52.4 |  |  | 44.9 | 38.8, 51.2 |
| **Education** | 2778 | 52,666 | - | - | 1821 | 26,278 | 49.9 | 46.2, 53.6 | 957 | 26,388 | 50.1 | 46.4, 53.8 |
| Elementary or lower |  |  | 28.7 | 25.6, 32.0 |  |  | 30.1 | 26.7, 33.8 |  |  | 27.2 | 22.8, 32.2 |
| High school |  |  | 55.1 | 51.4, 58.8 |  |  | 55.6 | 51.6, 59.6 |  |  | 54.6 | 48.7, 60.4 |
| Bachelor's degree or above |  |  | 16.2 | 13.1, 20.0 |  |  | 14.3 | 10.7, 18.8 |  |  | 18.2 | 13.4, 24.2 |
| **Area** | 2765 | 52,006 | - | - | 1815 | 26,037 |  |  | 950 | 25,969 |  |  |
| Rural |  |  | 23.9 | 21.3, 26.6 |  |  | 23.1 | 20.3, 26.3 |  |  | 24.6 | 21.1, 28.6 |
| Urban |  |  | 76.1 | 73.4, 78.7 |  |  | 76.9 | 73.7, 79.8 |  |  | 75.4 | 71.4, 78.9 |
| **Region** | 2778 | 52,666 | - | - | 1821 | 26,278 | 49.9 | 46.2, 53.6 | 957 | 26,388 | 50.1 | 46.4, 53.8 |
| North |  |  | 26.5 | 23.2, 30.0 |  |  | 25.9 | 22.2, 30.2 |  |  | 26.9 | 22.1, 32.4 |
| Center |  |  | 30.2 | 26.4, 34.3 |  |  | 28.3 | 24.1, 33.03 |  |  | 32.0 | 26.4, 38.2 |
| Mexico City and State of Mexico |  |  | 16.8 | 14.3, 19.6 |  |  | 18.6 | 14.6, 23.4 |  |  | 14.9 | 11.1, 19.9 |
| South |  |  | 26.5 | 23.6, 29.8 |  |  | 27.2 | 23.4, 31.1 |  |  | 26.2 | 22.1, 30.5 |
| **BMI^§^** | 2745 | 52,084 | - | - | 1801 | 26,038 |  |  | 944 | 26,046 |  |  |
| Normal |  |  | 24.3 | 21.7, 27.1 |  |  | 26.6 | 22.3, 31.5 |  |  | 21.9 | 19.03, 25.1 |
| Overweight |  |  | 40.6 | 37.2, 44.1 |  |  | 43.8 | 38.4, 49.3 |  |  | 37.4 | 33.5, 41.6 |
| Obesity |  |  | 35.1 | 31.6, 38.7 |  |  | 29.6 | 24.6, 35.1 |  |  | 40.7 | 36.5, 44.9 |
| **Blood Pressure^¶^** | 2716 | 51,591 | - | - | 1773 | 25,406 |  |  | 943 | 26,185 |  |  |
| Normal |  |  | 53.9 | 50.4, 57.3 |  |  | 63.9 | 59.7, 67.9 |  |  | 44.1 | 38.6, 49.8 |
| Elevated |  |  | 10.0 | 8.1, 12.3 |  |  | 6.0 | 4.4, 8.2 |  |  | 13.9 | 10.6, 17.9 |
| Stage 1 |  |  | 19.2 | 16.5,22.2 |  |  | 12.3 | 9.9, 15.1 |  |  | 25.9 | 21.2, 31.1 |
| Stage 2 |  |  | 7.6 | 6.0, 9.8 |  |  | 5.3 | 3.4, 8.04 |  |  | 9.9 | 7.2, 13.5 |
| Previously diagnosed HBP |  |  | 9.3 | 7.5, 11.6 |  |  | 12.5 | 9.8, 15.9 |  |  | 6.2 | 4.1, 9.2 |
| **Fasting glucose ^~~I~~^** | 2531 | 48,336 | - | - | 1663 | 24,444 |  |  | 868 | 23,891 |  |  |
| Normal |  |  | 67.7 | 64.7, 70.6 |  |  | 65.3 | 61.6, 68.8 |  |  | 70.3 | 65.2, 74.9 |
| Prediabetes |  |  | 22.6 | 19.7, 25.7 |  |  | 23.0 | 19.8, 26.6 |  |  | 22.1 | 17.7, 27.2 |
| Previously diagnosed Diabetes |  |  | 6.5 | 5.3, 8.01 |  |  | 8.5 | 6.3, 11.2 |  |  | 4.5 | 3.3, 6.4 |
| Survey finding Diabetes |  |  | 3.2 | 2.2, 4.5 |  |  | 3.2 | 2.3, 4.4 |  |  | 3.1 | 1.7, 5.7 |
| **Total cholesterol ^~~T~~^** | 2621 | 49,534 | - | - | 1735 | 25,042 |  |  | 886 | 24,491 |  |  |
| Normal |  |  | 66.4 | 62.9, 69.7 |  |  | 62.4 | 58.1, 66.6 |  |  | 70.5 | 63.0, 75.4 |
| Hypercholesterolemia |  |  | 33.6 | 30.3, 37.0 |  |  | 37.6 | 33.4, 41.9 |  |  | 29.5 | 24.7, 34.9 |
| **LDL-c ^**^** | 2425 | 45,979 | - | - | 1648 | 23,538 |  |  | 777 | 22,395 |  |  |
| Normal |  |  | 38.0 | 34.6, 41.5 |  |  | 34.7 | 30.9, 38.6 |  |  | 41.5 | 34.0, 47.1 |
| High LDL-c |  |  | 62.0 | 58.1, 65.4 |  |  | 65.3 | 61.4, 69.1 |  |  | 58.5 | 52.9, 63.9 |
| **HDL-c ^§§^** | 2621 | 49,534 |  |  | 1735 | 25,042 |  |  | 886 | 24,491 |  |  |
| Normal |  |  | 24.6 | 21.4, 28.2 |  |  | 20.6 | 17.1, 24.6 |  |  | 28.7 | 23.5,34.7 |
| Hypoalphalipoproteinemia |  |  | 75.4 | 71.9, 78.6 |  |  | 79.4 | 75.4, 81.9 |  |  | 71.3 | 65.4, 76.5 |
| **Triglycerides ^¶¶^** | 2621 | 49,534 | - | - | 1735 | 25,042 |  |  | 886 | 24, 491 |  |  |
| Normal |  |  | 43.2 | 39.6, 46.9 |  |  | 49.9 | 45.5, 4.3 |  |  | 36.4 | 31.1, 41.9 |
| Hypertriglyceridemia |  |  | 56.8 | 53.2, 60.4 |  |  | 50.1 | 45.7, 54.5 |  |  | 63.7 | 58.0, 68.9 |
| **Previous medical diagnosis ^~~II~~^** | 2735 | 52,059 | - | - | 1795 | 26,111 |  |  | 940 | 25,948 |  |  |
| Cerebrovascular disease diagnosis |  |  | 0.41 | 0.22, 0.8 |  |  | 0.6 | 0.30, 1.3 |  |  | 0.2 | 0.06, 0.55 |
|  | 2763 | 52,342 | - | - | 1814 | 26,211 |  |  | 949 | 26,130 |  |  |
| Coronary heart disease |  |  | 1.7 | 1.04, 2.74 |  |  | 1.3 | 0.76, 2.1 |  |  | 2.1 | 1.03, 4.3 |

Estimates were adjusted for complex survey design. ^§^ Body mass index (BMI): <25 kg/m^2^ (normal); 25-29.9 kg/m^2^ (overweight); ≥30 kg/m^2^ (obesity). ^¶^ Blood pressure (mm Hg): normal (<120/80); elevated (systolic between 120-129 and diastolic <80); stage 1 (systolic between 130-139 or diastolic between 80-89); stage 2 (systolic at least 140 or diastolic at least 90). ^~~I~~^ Fasting glucose: prediabetes (fasting glucose ≥100 y <126 mg/dL or HbA1c ≥5.7 and <6.5%); survey finding (fasting glucose ≥126 mg/dL or HbA1c ≥6.5%). ^~~T~~^ High total cholesterol levels: ≥200 mg/dL. ^**^ High LDL-c levels: ≥100 mg/dL. ^§§^ Low HDL-c levels (hypoalphalipoproteinemia): <40 mg/dL. ^¶¶^ High triglycerides levels: ≥150 mg/dL. ^~~I~~I^ Self-report of previous medical diagnosis.
