# Supplementary material for: Sodium and potassium excretion and its association with cardiovascular disorders in Mexican adults
Source: Front Nutr. 2024 Jun 24;11:1395016. doi: 10.3389/fnut.2024.1395016 (PMC11228292; doi:10.3389/fnut.2024.1395016)
Supplement: Supplementary file 2 [file Table_2.DOCX]

**Supplementary Table 2.** Distribution of sodium, potassium excretion and sodium-potassium ratio estimated from spot urine sample of Mexican adults 20-59 years. ENSANUT-2016.

|  | **Sodium excretion (mg/day)** | | | **Potassium excretion (mg/day)** | | | **Na-K ratio** | | |
| --- | --- | --- | --- | --- | --- | --- | --- | --- | --- |
|  | **n** | **mean** | **95% CI** | **n** | **mean** | **95% CI** | **n** | **mean** | **95% CI** |
| **National** | 2772 | 3354.4 | 3278.9, 3429.8 | 2799 | 1440.7 | 1412.0, 1469.4 | 2771 | 2.4 | 2.3, 2.5 |
| **Sex** | 2772 |  |  | 2799 |  |  | 2771 |  |  |
| Women |  | 3273.9 | 3194.8, 3353.1 |  | 1436.4 | 1403.3, 1469.5 |  | 2.4 | 2.3, 2.4 |
| Men |  | 3442.9 | 3318.4, 3567.3 |  | 1445.4 | 1400.1, 1490.7 |  | 2.5 | 2.4, 2.5 |
| **Age (years)** | 2772 |  |  | 2799 |  |  | 2771 |  |  |
| 20-29 |  | 3425.6 | 3288.3, 3562.8 |  | 1414.2 | 1368.1, 1460.3 |  | 2.5 | 2.4, 2.6 |
| 30-39 |  | 3371.3 | 3208.8, 3533.8 |  | 1381.3 | 1329.9, 1432.6 |  | 2.5 | 2.4, 2.6 |
| 40-49 |  | 3275.1 | 3171.0, 3379.3 |  | 1460.6 | 1414.6, 1506.7 |  | 2.3 | 2.2, 2.4 |
| 50-59 |  | 3311.3 | 3129.9, 3492.6 |  | 1557.0 | 1494.0, 1619.9 |  | 2.2 | 2.1, 2.3 |
| **Socioeconomic index** | 2771 |  |  | 2798 |  |  | 2770 |  |  |
| Low |  | 3265.4 | 3173.5, 3357.3 |  | 1377.0 | 1336.8, 1417.3 |  | 2.4 | 2.4, 2.5 |
| Middle |  | 3363.0 | 3239.5, 3486.6 |  | 1439.8 | 1398.6, 1480.9 |  | 2.4 | 2.3, 2.5 |
| High |  | 3387.8 | 3259.5, 3516.1 |  | 1469.4 | 1419.7, 1519.0 |  | 2.4 | 2.3, 2.5 |
| **Education** | 2771 |  |  | 2798 |  |  | 2770 |  |  |
| Elementary or lower |  | 3344.4 | 3257.5, 3431.3 |  | 1462.6 | 1420.2, 1505.1 |  | 2.4 | 2.3, 2.5 |
| High school |  | 3355.5 | 3249.6, 3461.4 |  | 1436.0 | 1397.0, 1475.0 |  | 2.4 | 2.3, 2.5 |
| Bachelor's degree or above |  | 3367.5 | 3171.1, 3563.9 |  | 1417.9 | 1347.2, 1488.7 |  | 2.4 | 2.3, 2.5 |
| **Area** | 2771 |  |  | 2798 |  |  | 2770 |  |  |
| Rural |  | 3294.0 | 3209.5, 3378.4 |  | 1419.1 | 1388.4, 1449.9 |  | 2.4 | 2.3, 2.5 |
| Urban |  | 3373.9 | 3182.6, 3565.1 |  | 1454.5 | 1383.9, 1525.0 |  | 2.4 | 2.3, 2.5 |
| **Region** | 2772 |  |  | 2799 |  |  | 2771 |  |  |
| North |  | 3481.0 | 3329.2, 3632.8 |  | 1520.3 | 1462.8, 1577.9 |  | 2.4 | 2.3, 2.5 |
| Center |  | 3383.5 | 3232.3, 3534.8 |  | 1433.9 | 1378.3, 1489.5 |  | 2.4 | 2.3, 2.6 |
| Mexico City and State of Mexico |  | 3313.5 | 3093.1, 3533.9 |  | 1453.0 | 1367.4, 1538.5 |  | 2.3 | 2.2, 2.5 |
| South |  | 3213.1* | 3132.0, 3294.2 |  | 1355.8 | 1325.6, 1386.1 |  | 2.4 | 2.4, 2.5 |
| **BMI^§^** | 2768 |  |  | 2794 |  |  | 2767 |  |  |
| Normal |  | 3113.7 | 3014.0, 3213.4 |  | 1333.1 | 1285.9, 1380.3 |  | 2.4 | 2.3, 2.5 |
| Overweight |  | 3323.4 | 3193.0, 3453.8 |  | 1417.7 | 1366.6, 1468.8 |  | 2.4 | 2.3, 2.5 |
| Obesity |  | 3553.9 | 3424.6, 3683.2 |  | 1538.6 | 1504.6, 1572.6 |  | 2.4 | 2.3, 2.5 |
| **Blood Pressure^¶^** | 2709 |  |  | 2736 |  |  | 2708 |  |  |
| Normal |  | 3296.2 | 3196.3, 3396.1 |  | 1396.5 | 1357.7, 1435.3 |  | 2.4 | 2.4, 2.5 |
| Elevated |  | 3542.4 | 3285.7, 3799.2 |  | 1465.9 | 1382.4, 1549.5 |  | 2.5 | 2.3, 2.6 |
| Stage 1 |  | 3427.9 | 3234.6, 3621.2 |  | 1490.5 | 1439.5, 1541.6 |  | 2.4 | 2.2, 2.5 |
| Stage 2 |  | 3369.2 | 3097.1, 3641.3 |  | 1526.4 | 1435.2, 1617.6 |  | 2.2 | 2.1, 2.4 |
| Previously diagnosed HBP |  | 3296.4 | 3175.1, 3417.6 |  | 1534.6 | 1470.7, 1598.5 |  | 2.2* | 2.1, 2.3 |
| **Fasting glucose ^~~I~~^** | 2478 |  |  | 2502 |  |  | 2477 |  |  |
| Normal |  | 3299.4 | 3195.4, 3403.5 |  | 1419.9 | 1383.2, 1456.5 |  | 2.4 | 2.3, 2.5 |
| Prediabetes |  | 3524.2 | 3367.7, 3680.7 |  | 1473.5 | 1423.1, 1524.0 |  | 2.5 | 2.4, 2.5 |
| Previously diagnosed Diabetes |  | 3318.8 | 3075.7, 3561.9 |  | 1534.6 | 1460.4, 1608.7 |  | 2.2 | 2.1, 2.3 |
| Survey finding Diabetes |  | 3451.6 | 3125.0, 3778.3 |  | 1492.8 | 1368.6, 1617.0 |  | 2.4 | 2.2, 2.5 |
| **Cholesterol ^~~T~~^** | 2567 |  |  | 2592 |  |  | 2566 |  |  |
| Normal |  | 3417.8 | 3339.2, 3496.5 |  | 1422.2 | 1389.7, 1454.8 |  | 2.5 | 2.4, 2.5 |
| Hypercholesterolemia |  | 3249.1 | 3093.9, 3404.3 |  | 1473.1 | 1415.1, 1531.2 |  | 2.3* | 2.1, 2.4 |
| **LDL-c ^**^** | 2372 |  |  | 2397 |  |  | 2371 |  |  |
| Normal |  | 3417.5 | 3315.6, 3519.4 |  | 1434.4 | 1389.9, 1478.9 |  | 2.5 | 2.4, 2.5 |
| High LDL-c |  | 3289.36 | 3182.4, 3396.4 |  | 1441.0 | 1403.2, 1478.9 |  | 2.4 | 2.3, 2.4 |
| **HDL-c ^§§^** | 2567 |  |  | 2592 |  |  | 2566 |  |  |
| Normal |  | 3227.3 | 3111.3, 3343.4 |  | 1444.3 | 1397.6, 1491.0 |  | 2.3 | 2.2, 2.4 |
| Hypoalphalipoproteinemia |  | 3439.4 | 3336.3, 3542.6 |  | 1439.1 | 1402.5, 1475.6 |  | 2.5 | 2.4, 2.5 |
| **Triglycerides ^¶¶^** | 2567 |  |  | 2592 |  |  | 2566 |  |  |
| Normal |  | 3338.8 | 3225.7, 3451.8 |  | 1411.7 | 1363.5, 1460.0 |  | 2.5 | 2.3, 2.6 |
| Hypertriglyceridemia |  | 3367.8 | 3254.8, 3480.8 |  | 1465.3 | 1431.4, 1499.3 |  | 2.4 | 2.3, 2.4 |
| **Altered renal function ^h^** | 2561 |  |  | 2586 |  |  | 2560 |  |  |
| Normal |  | 3363.0 | 3280.5, 3445.4 |  | 1432.0 | 1401.0, 1463.0 |  | 2.4 | 2.4, 2.5 |
| Mildly reduced |  | 3291.7 | 3101.3, 3482.0 |  | 1547.4* | 1437.6, 1657.2 |  | 2.2 | 2.0, 2.4 |
| Moderately reduced |  | 2760.2* | 2259.4, 3261.0 |  | 1317.1 | 1090.9, 1543.3 |  | 2.0* | 1.9, 2.2 |
| **Cerebrovascular disease diagnosis** | 2729 |  |  | 2755 |  |  | 2728 |  |  |
| Negative |  | 3348.2 | 3272.3, 3424.0 |  | 1441.5 | 1412.9, 1470.2 |  | 2.4 | 2.3, 2.5 |
| Positive |  | 3861.4 | 3048.2, 4674.7 |  | 1529.2 | 1301.0, 1757.3 |  | 2.5 | 2.2, 2.9 |
| **Coronary heart disease diagnosis** | 2756 |  |  | 2783 |  |  | 2755 |  |  |
| Negative |  | 3346.3 | 3270.0, 3422.7 |  | 1441.7 | 1412.8, 1470.6 |  | 2.4 | 2.3, 2.5 |
| Positive |  | 3614.5 | 3235.8, 3993.2 |  | 1407.2 | 1249.9, 1564.5 |  | 2.6 | 2.3, 3.0 |

^*^ National Health and Nutrition Survey 2016. Estimates were adjusted for complex survey design. ^§^ Body mass index (BMI): <25 kg/m^2^ (normal); 25-29.9 kg/m^2^ (overweight); ≥30 kg/m^2^ (obesity). ^¶^ Blood pressure (mm Hg): normal (<120/80); elevated (systolic between 120-129 and diastolic <80); stage 1 (systolic between 130-139 or diastolic between 80-89); stage 2 (systolic at least 140 or diastolic at least 90).^~~I~~^ Fasting glucose: prediabetes (fasting glucose ≥100 y <126 mg/dL or HbA1c ≥5.7 and <6.5%); survey finding (fasting glucose ≥126 mg/dL or HbA1c ≥6.5%). ^~~T~~^ High total cholesterol levels: ≥200 mg/dL. ^**^ High LDL-c levels: ≥100 mg/dL. ^§§^ Low HDL-c levels (hypoalphalipoproteinemia): <40 mg/dL. ^¶¶^ High triglycerides levels: ≥150 mg/dL. ^~~TT~~^ Altered renal function: glomerular filtration rate (eGFR) ml/min/1.73m^2^ normal (≥90); mildly reduced (60-89); moderately reduced (30-59); severely reduced (15-29). The mean (95% CIs) of variables were tabulated across urinary Na excretion, K excretion and Na-K ratio and compared using ANOVA tests.
